# Supplementary material for: Macrophage-Immunomodulatory Actions of Bovine Whey Protein Isolate, Glycomacropeptide, and Their In Vitro and In Vivo Digests
Source: Nutrients. 2023 Nov 28;15(23):4942. doi: 10.3390/nu15234942 (PMC10707750; doi:10.3390/nu15234942)
Supplement: Supplementary file 1 [file nutrients-15-04942-s001.zip › nutrients-2681511-supplementary.pdf]

Supplementary Results

Table S1. Cell viability was assessed by the MTT assay. The results were expressed as % of control (media only) and represent the means of three independent experiments.

Treatment	Cell Viability (% of Control) [a]
Inducer only (LPS)	96.0 ± 4.8
WPI[b]1000	91.9 ± 4.8
WPI 100	95.0 ± 5.4
WPI 10	98.1 ± 4.1
WPID[c] 1000	97.2 ± 3.6
WPID 100	97.2 ± 2.8
WPID 10	92.6 ± 4.1
GMP[d] 1000	94.1 ± 9.6
GMP 100	96.6 ± 2.3
GMP 10	96.0 ± 5.6
GMPD[e] 1000	92.9 ± 7.3
GMPD 100	93.6 ± 3.2
GMPD 10	90.0 ± 3.3
WPIINT[f] 1000	94.7 ± 3.5
WPIINT100	94.1 ± 4.9
WPIINT10	100.5 ± 5.8

[a] One-sided t-tests were conducted to test if there are significant differences between each treatment from the LPS-only control ($P < 0.05$). No significant different was found between each treatment and LPS-only control. n = 6 technical replicates (3 days of cell experiment replication, duplicate wells on each day).
[b] Whey protein isolate (WPI)
[c] In vitro-digested whey protein isolate (WPID)
[d] Glycomacropeptide (GMP)
[e] In vitro-digested glycomacropeptide (GMPD)
[f] Intestinal-digested whey protein isolate (WPIINT)

Table S2. Concentrations of cytokines in cells based on treatment.

Treatment	TNF-α (pg/mL)[a]	IL-1β (pg/mL)
Blank cell	33.6±11.9	67.4±10.7
Inducer only (LPS)	216.3 ± 19.3	313.3±47.7
WPI[b] 1000	233.9±18.1	132.2±16.2
WPI 100	234.5±22.5	146.0±17.4
WPI 10	218.4±11.8	178.8±26.3
WPID[c] 1000	115.5±17.3	152.6±14.6
WPID 100	149.4±20.9	157.5±22.8
WPID 10	178.1±22.2	171.9±22.5
GMP[d] 1000	276.3±39.2	492.2±83.8
GMP 100	262.6±19.2	430.2±63.1

GMP 10	89.5±21.6	310.8±46.1
GMPDe 1000	109.0±11.3	151.2±14.6
GMPD 100	157.1±14.4	114.7±17.7
GMPD 10	58.6±26.9	212.5±30.2
WPIINT[f] 1000	58.6±26.9	421.7±57.6
WPIINT 100	60.6±28.3	327.0±50.5
WPIINT 10	63.5±2.9	160.8±14.9

[a] n = 6 technical replicates (3 days of cell experiment replication, duplicate wells on each day).
[b] Whey protein isolate (WPI)
[c] In vitro-digested whey protein isolate (WPID)
[d] Glycomacropeptide (GMP)
[e] In vitro-digested glycomacropeptide (GMPD)
[f] Intestinal-digested whey protein isolate (WPIINT)

Table S3. Two-sided t-tests were performed to determine whether each cytokine concentration (TNF-α and IL-1β) in each treatment (WPI, GMP, WPID, GMPD, WPIINT at each dose (10, 100 and 1,000 μg/mL)) were significantly different from the LPS-only control using GraphPad Prism software (version 8.2).

	P value t-test compared to control	
	TNF-α[a]	IL-1β
WPI 1000[b]	0.1332	<0.0001
WPI 100	0.1639	<0.0001
WPI 10	0.8233	0.0001
WPID 1000[c]	<0.0001	<0.0001
WPID 100	0.0002	<0.0001
WPID 10	0.0098	<0.0001
GMP 1000[d]	0.0071	0.0011
GMP 100	0.0335	0.0047
GMP 10	0.8162	0.9293

GMPD 1000[e]	<0.0001	<0.0001
GMPD 100	<0.0001	<0.0001
GMPD 10	0.0001	0.0014
WPIINT 1000[f]	<0.0001	0.0053
WPIINT 100	<0.0001	0.6412
WPIINT 10	<0.0001	<0.0001

[a] n = 6 technical replicates (3 days of cell experiment replication, duplicate wells on each day).
[b] Whey protein isolate (WPI)
[c] In vitro-digested whey protein isolate (WPID)
[d] Glycomacropeptide (GMP)
[e] In vitro-digested glycomacropeptide (GMPD)
[f] Intestinal-digested whey protein isolate (WPIINT)

Digested	Concentration	-Digested	-Concentration	Difference	Std Error	t Ratio	Prob>|t|	Lower 95%	Upper 95%
No	10	No	100	-0.090000	0.0636479	-1.41	0.8861	-0.298034	0.118034
No	10	No	1000	-0.091667	0.0636479	-1.44	0.8755	-0.299701	0.116367
No	10	Simulated	10	0.226667	0.0636479	3.56	0.0236*	0.018633	0.434701
No	10	Simulated	100	0.381667	0.0636479	6.00	<.0001*	0.173633	0.589701
No	10	Simulated	1000	0.571667	0.0636479	8.98	<.0001*	0.363633	0.779701
No	10	Intestinal	10	0.853333	0.0636479	13.41	<.0001*	0.645299	1.061367
No	10	Intestinal	100	0.875000	0.0636479	13.75	<.0001*	0.666966	1.083034
No	10	Intestinal	1000	0.885000	0.0636479	13.90	<.0001*	0.676966	1.093034
No	100	No	1000	-0.001667	0.0636479	-0.03	1.0000	-0.209701	0.206367
No	100	Simulated	10	0.316667	0.0636479	4.98	0.0004*	0.108633	0.524701
No	100	Simulated	100	0.471667	0.0636479	7.41	<.0001*	0.263633	0.679701
No	100	Simulated	1000	0.661667	0.0636479	10.40	<.0001*	0.453633	0.869701
No	100	Intestinal	10	0.943333	0.0636479	14.82	<.0001*	0.735299	1.151367
No	100	Intestinal	100	0.965000	0.0636479	15.16	<.0001*	0.756966	1.173034
No	100	Intestinal	1000	0.975000	0.0636479	15.32	<.0001*	0.766966	1.183034
No	1000	Simulated	10	0.318333	0.0636479	5.00	0.0003*	0.110299	0.526367
No	1000	Simulated	100	0.473333	0.0636479	7.44	<.0001*	0.265299	0.681367
No	1000	Simulated	1000	0.663333	0.0636479	10.42	<.0001*	0.455299	0.871367
No	1000	Intestinal	10	0.945000	0.0636479	14.85	<.0001*	0.736966	1.153034
No	1000	Intestinal	100	0.966667	0.0636479	15.19	<.0001*	0.758633	1.174701
No	1000	Intestinal	1000	0.976667	0.0636479	15.34	<.0001*	0.768633	1.184701
Simulated	10	Simulated	100	0.155000	0.0636479	2.44	0.2925	-0.053034	0.363034
Simulated	10	Simulated	1000	0.345000	0.0636479	5.42	<.0001*	0.136966	0.553034
Simulated	10	Intestinal	10	0.626667	0.0636479	9.85	<.0001*	0.418633	0.834701
Simulated	10	Intestinal	100	0.648333	0.0636479	10.19	<.0001*	0.440299	0.856367
Simulated	10	Intestinal	1000	0.658333	0.0636479	10.34	<.0001*	0.450299	0.866367
Simulated	100	Simulated	1000	0.190000	0.0636479	2.99	0.0976	-0.018034	0.398034
Simulated	100	Intestinal	10	0.471667	0.0636479	7.41	<.0001*	0.263633	0.679701
Simulated	100	Intestinal	100	0.493333	0.0636479	7.75	<.0001*	0.285299	0.701367
Simulated	100	Intestinal	1000	0.503333	0.0636479	7.91	<.0001*	0.295299	0.711367
Simulated	1000	Intestinal	10	0.281667	0.0636479	4.43	0.0020*	0.073633	0.489701
Simulated	1000	Intestinal	100	0.303333	0.0636479	4.77	0.0007*	0.095299	0.511367
Simulated	1000	Intestinal	1000	0.313333	0.0636479	4.92	0.0004*	0.105299	0.521367
Intestinal	10	Intestinal	100	0.021667	0.0636479	0.34	1.0000	-0.186367	0.229701
Intestinal	10	Intestinal	1000	0.031667	0.0636479	0.50	0.9999	-0.176367	0.239701
Intestinal	100	Intestinal	1000	0.010000	0.0636479	0.16	1.0000	-0.198034	0.218034

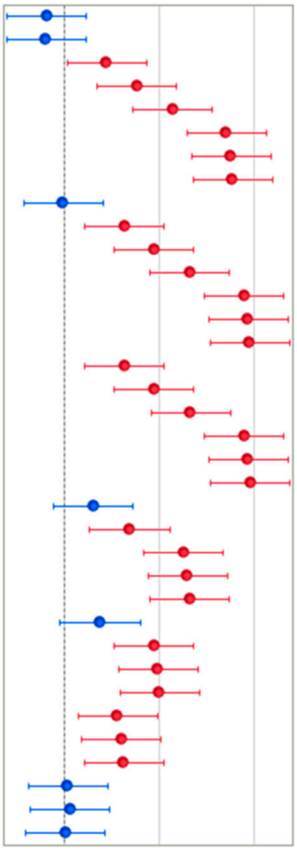

Figure S1. The significance of the differences in percent change of TNF-α from the LPS-only control among WPI, WPID and WPIINT at each concentration were assessed using multi-factor ANOVA followed by Tukey's HSD tests, using JMP software (version 17). n = 6 technical replicates (3 days of cell experiment replication, duplicate wells on each day).

Digested	Concentration	-Digested	-Concentration	Difference	Std Error	t Ratio	Prob>|t|	Lower 95%	Upper 95%
No	10	No	100	0.13000	0.0342030	3.80	0.0123*	0.01821	0.24179
No	10	No	1000	0.19167	0.0342030	5.60	<.0001*	0.07987	0.30346
No	10	Simulated	10	0.02000	0.0342030	0.58	0.9996	-0.09179	0.13179
No	10	Simulated	100	0.08833	0.0342030	2.58	0.2245	-0.02346	0.20013
No	10	Simulated	1000	0.10667	0.0342030	3.12	0.0718	-0.00513	0.21846
No	10	Intestinal	10	0.07000	0.0342030	2.05	0.5215	-0.04179	0.18179
No	10	Intestinal	100	-0.60000	0.0342030	-17.54	<.0001*	-0.71179	-0.48821
No	10	Intestinal	1000	-0.99000	0.0342030	-28.94	<.0001*	-1.10179	-0.87821
No	100	No	1000	0.06167	0.0342030	1.80	0.6800	-0.05013	0.17346
No	100	Simulated	10	-0.11000	0.0342030	-3.22	0.0568	-0.22179	0.00179
No	100	Simulated	100	-0.04167	0.0342030	-1.22	0.9478	-0.15346	0.07013
No	100	Simulated	1000	-0.02333	0.0342030	-0.68	0.9988	-0.13513	0.08846
No	100	Intestinal	10	-0.06000	0.0342030	-1.75	0.7104	-0.17179	0.05179
No	100	Intestinal	100	-0.73000	0.0342030	-21.34	<.0001*	-0.84179	-0.61821
No	100	Intestinal	1000	-1.12000	0.0342030	-32.75	<.0001*	-1.23179	-1.00821
No	1000	Simulated	10	-0.17167	0.0342030	-5.02	0.0003*	-0.28346	-0.05987
No	1000	Simulated	100	-0.10333	0.0342030	-3.02	0.0900	-0.21513	0.00846
No	1000	Simulated	1000	-0.08500	0.0342030	-2.49	0.2681	-0.19679	0.02679
No	1000	Intestinal	10	-0.12167	0.0342030	-3.56	0.0239*	-0.23346	-0.00987
No	1000	Intestinal	100	-0.79167	0.0342030	-23.15	<.0001*	-0.90346	-0.67987
No	1000	Intestinal	1000	-1.18167	0.0342030	-34.55	<.0001*	-1.29346	-1.06987
Simulated	10	Simulated	100	0.06833	0.0342030	2.00	0.5533	-0.04346	0.18013
Simulated	10	Simulated	1000	0.08667	0.0342030	2.53	0.2456	-0.02513	0.19846
Simulated	10	Intestinal	10	0.05000	0.0342030	1.46	0.8663	-0.06179	0.16179
Simulated	10	Intestinal	100	-0.62000	0.0342030	-18.13	<.0001*	-0.73179	-0.50821
Simulated	10	Intestinal	1000	-1.01000	0.0342030	-29.53	<.0001*	-1.12179	-0.89821
Simulated	100	Simulated	1000	0.01833	0.0342030	0.54	0.9998	-0.09346	0.13013
Simulated	100	Intestinal	10	-0.01833	0.0342030	-0.54	0.9998	-0.13013	0.09346
Simulated	100	Intestinal	100	-0.68833	0.0342030	-20.12	<.0001*	-0.80013	-0.57654
Simulated	100	Intestinal	1000	-1.07833	0.0342030	-31.53	<.0001*	-1.19013	-0.96654
Simulated	1000	Intestinal	10	-0.03667	0.0342030	-1.07	0.9752	-0.14846	0.07513
Simulated	1000	Intestinal	100	-0.70667	0.0342030	-20.66	<.0001*	-0.81846	-0.59487
Simulated	1000	Intestinal	1000	-1.09667	0.0342030	-32.06	<.0001*	-1.20846	-0.98487
Intestinal	10	Intestinal	100	-0.67000	0.0342030	-19.59	<.0001*	-0.78179	-0.55821
Intestinal	10	Intestinal	1000	-1.06000	0.0342030	-30.99	<.0001*	-1.17179	-0.94821
Intestinal	100	Intestinal	1000	-0.39000	0.0342030	-11.40	<.0001*	-0.50179	-0.27821

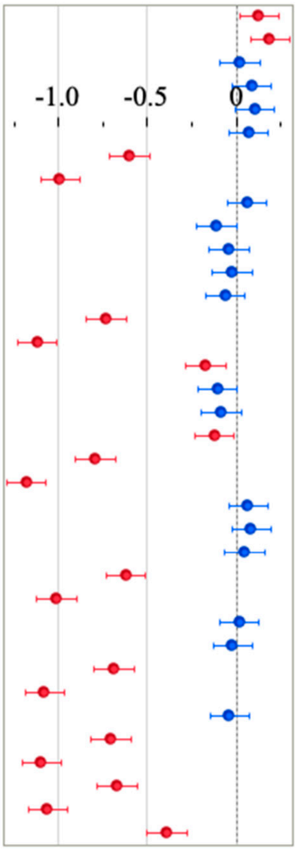

Figure S2. The significance of the differences in percent change of IL-1β from the LPS-only control among WPI, WPID and WPIINT at each concentration were assessed using multi-factor ANOVA followed by Tukey's HSD tests, using JMP software (version 17). n = 6 technical replicates (3 days of cell experiment replication, duplicate wells on each day).

Digested	Concentration	-Digested	-Concentration	Difference	Std Error	t Ratio	Prob>|t|	Lower 95%	Upper 95%
No	10	No	100	-0.27167	0.0655120	-4.15	0.0037*	-0.472386	-0.07095
No	10	No	1000	-0.35167	0.0655120	-5.37	0.0002*	-0.552386	-0.15095
No	10	Simulated	10	0.31000	0.0655120	4.73	0.0008*	0.109281	0.51072
No	10	Simulated	100	0.57500	0.0655120	8.78	<.0001*	0.374281	0.77572
No	10	Simulated	1000	0.68667	0.0655120	10.48	<.0001*	0.485948	0.88739
No	100	No	1000	-0.08000	0.0655120	-1.22	0.8228	-0.280719	0.12072
No	100	Simulated	10	0.58167	0.0655120	8.88	<.0001*	0.380948	0.78239
No	100	Simulated	100	0.84667	0.0655120	12.92	<.0001*	0.645948	1.04739
No	100	Simulated	1000	0.95833	0.0655120	14.63	<.0001*	0.757614	1.15905
No	1000	Simulated	10	0.66167	0.0655120	10.10	<.0001*	0.460948	0.86239
No	1000	Simulated	100	0.92667	0.0655120	14.14	<.0001*	0.725948	1.12739
No	1000	Simulated	1000	1.03833	0.0655120	15.85	<.0001*	0.837614	1.23905
Simulated	10	Simulated	100	0.26500	0.0655120	4.05	0.0047*	0.064281	0.46572
Simulated	10	Simulated	1000	0.37667	0.0655120	5.75	<.0001*	0.175948	0.57739
Simulated	100	Simulated	1000	0.11167	0.0655120	1.70	0.5407	-0.089052	0.31239

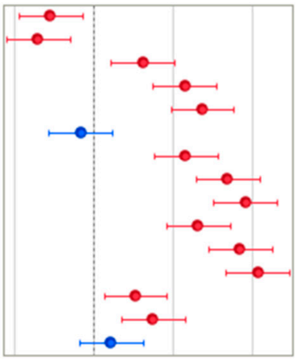

Figure S3. The significance of the differences in percent change of TNF-α from the LPS-only control among GMP and GMPD at each concentration were assessed using multi-factor ANOVA followed by Tukey's HSD tests, using JMP software (version 17). n = 6 technical replicates (3 days of cell experiment replication, duplicate wells on each day).

Digested	Concentration	-Digested	-Concentration	Difference	Std Error	t Ratio	Prob>|t|	Lower 95%	Upper 95%
No	10	No	100	-0.48167	0.0657943	-7.32	<.0001*	-0.68325	-0.28008
No	10	No	1000	-0.73167	0.0657943	-11.12	<.0001*	-0.93325	-0.53008
No	10	Simulated	10	0.41000	0.0657943	6.23	<.0001*	0.20842	0.61158
No	10	Simulated	100	0.80667	0.0657943	12.26	<.0001*	0.60508	1.00825
No	10	Simulated	1000	0.65667	0.0657943	9.98	<.0001*	0.45508	0.85825
No	100	No	1000	-0.25000	0.0657943	-3.80	0.0088*	-0.45158	-0.04842
No	100	Simulated	10	0.89167	0.0657943	13.55	<.0001*	0.69008	1.09325
No	100	Simulated	100	1.28833	0.0657943	19.58	<.0001*	1.08675	1.48992
No	100	Simulated	1000	1.13833	0.0657943	17.30	<.0001*	0.93675	1.33992
No	1000	Simulated	10	1.14167	0.0657943	17.35	<.0001*	0.94008	1.34325
No	1000	Simulated	100	1.53833	0.0657943	23.38	<.0001*	1.33675	1.73992
No	1000	Simulated	1000	1.38833	0.0657943	21.10	<.0001*	1.18675	1.58992
Simulated	10	Simulated	100	0.39667	0.0657943	6.03	<.0001*	0.19508	0.59825
Simulated	10	Simulated	1000	0.24667	0.0657943	3.75	0.0100*	0.04508	0.44825
Simulated	100	Simulated	1000	-0.15000	0.0657943	-2.28	0.2367	-0.35158	0.05158

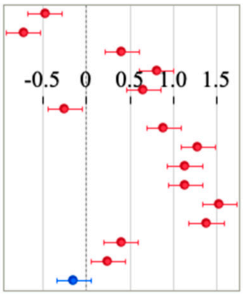

Figure S4. The significance of the differences in percent change of IL-1β from the LPS-only control among GMP and GMPD at each concentration were assessed using multi-factor ANOVA followed by Tukey's HSD tests, using JMP software (version 17). n = 6 technical replicates (3 days of cell experiment replication, duplicate wells on each day).
